# Identification of sentinel lymph nodes by contrast‐enhanced ultrasonography with Sonazoid in patients with breast cancer: a feasibility study in three hospitals

**DOI:** 10.1002/cam4.1142

**Published:** 2017-08-01

**Authors:** Kenzo Shimazu, Toshikazu Ito, Kumiko Uji, Tomohiro Miyake, Toyokazu Aono, Kazuyoshi Motomura, Yasuto Naoi, Atsushi Shimomura, Masafumi Shimoda, Naofumi Kagara, Seung Jin Kim, Shinzaburo Noguchi

**Affiliations:** ^1^ Department of Breast and Endocrine Surgery Osaka University Graduate School of Medicine 2‐2 Yamadaoka Suita‐shi Osaka 565‐0871 Japan; ^2^ Department of Surgery Rinku General Medical Center Osaka Japan; ^3^ Department of Breast Surgery Osaka General Medical Center Osaka Japan

**Keywords:** Breast cancer, contrast‐enhanced ultrasonography, fine needle aspiration cytology, sentinel lymph node biopsy, Sonazoid

## Abstract

The aim of this prospective study was to evaluate the feasibility of periareolar injection of the contrast agent Sonazoid (SNZ) followed by ultrasonography (US) for the identification of sentinel lymph node (SLN) in breast cancer patients with clinically negative node. Patients (*n* = 100) with T1‐2N0M0 breast cancer received a periareolar injection of SNZ followed by US to identify contrast‐enhanced SLN. Each contrast‐enhanced SLN underwent fine needle aspiration cytology (FNAC) followed by SLN biopsy with a conventional method using blue dye and/or radiocolloid (B/R). In almost all cases, contrast‐enhanced lymphatic vessels were clearly visualized by US soon after the periareolar injection of SNZ and the SLNs were easily identified with an identification rate of 98% (98/100) for SNZ and 100% (100/100) for B/R. The number of SLNs identified by SNZ (SNZ‐SLN) (mean per patient, 1.52) was significantly lower than that identified by B/R (B/R‐SLN) (2.19) (*P* < 0.0001). Twenty‐five patients with positive SLNs had at least one positive SNZ‐SLN. On a node‐by‐node basis, sensitivity, specificity, and accuracy of FNAC for SNZ‐SLNs (*n* = 149) were 33.3%, 99.2%, and 85.9%, respectively. Identification of SLN by periareolar injection of SNZ is a technically simple method with an identification rate as high as 98%. SNZ‐SLN thus seems to be a good target for FNAC, but sensitivity of FNAC for SNZ‐SLNs needs to be improved.

## Introduction

Axillary lymph node status remains an important prognostic factor, providing valuable information for decision making regarding adjuvant therapy for breast cancer patients. Many studies have reported that sentinel lymph node biopsy (SLNB) can accurately predict axillary lymph node (ALN) status 1, 2, 3, 4, 5, and has therefore replaced axillary lymph node dissection (ALND) for clinically node‐negative patients and also for selected node‐positive patients. When the sentinel lymph node (SLN) is negative for metastasis, the patients can be spared ALND, which is associated with comorbidities such as paresthesia (31–74%), lymphedema (4–57%), seroma (14–16%), and limited shoulder motion (24–75%) 6, 7, 8. Although these comorbidities occur significantly less frequently in patients treated with SLNB, they still account for a considerable proportion (7–41%) of such patients 6, 9. Moreover, the blue dye often used for SLNB can cause severe anaphylaxis (0.6–2.7%) 10. In view of the fact that the SLNs of about 70–80% of the patients treated with SLNB are eventually found to be metastasis negative, it is of major clinical importance to develop a less invasive means for the determination of SLN status with an accuracy comparable to that of SLNB.

Various diagnostic imaging modalities, including computed tomography (CT), magnet resonance imaging (MRI), and ultrasonography (US), have been used to evaluate axillary node status 11, 12. These noninvasive modalities have proven to be moderately accurate for the determination of ALN status, but their accuracy is not high enough for SLNB to be replaced. Recently, however, a new, noninvasive means for the detection of SLNs has been introduced, which enables real‐time visualization of lymphatic flow and SLNs by using contrast‐enhanced ultrasonography (CEUS) with periareolarly injected Sonazoid (SNZ) (Daiichi‐Sankyo, Tokyo, Japan) 13. SNZ is a second‐generation ultrasound contrast agent made from lipid‐stabilized perfluorobutane microbubbles which remain chemically stable in lymphatic vessels, thus making real‐time lymphatic flow imaging feasible for a comparatively long time 14, 15. Omoto et al. recently reported their preliminary finding that SLNs could be detected in 75% of 20 breast cancer patients who underwent periareolar injection of SNZ followed by US of the axilla 13. Since the SLNs identified by SNZ are amenable to fine needle aspiration cytology (FNAC) or core needle biopsy (CNB), it may be possible to detect SLN metastasis without SLNB. In fact, two studies have been published which primarily evaluate CNB for detection of metastasis in SLNs identified with a different contrast agent, Sonovue (Bracco, Milan, Italy), with reported sensitivities of CNB on a per‐patient basis of 65% 16 and 53% 17.

These results for the identification of SLN by periareolar injection of a contrast agent as well as for that of CNB of such SLNs are of major interest and seem to be promising as constituting a less invasive method than SLNB for detection of SLN metastasis. The aim of our study was therefore to evaluate the feasibility of identification of SLNs by means of SNZ as well as the diagnostic accuracy of FNAC as a less invasive procedure than CNB for breast cancer patients with clinically negative axillary nodes. We hypothesized that, if the sensitivity of FNAC is found to be high enough, SLNB can be avoided for cases with negative FNAC.

## Patients and Methods

Female patients with T1‐2 N0 breast cancer were enrolled in this prospective study at Osaka University Hospital, Rinku General Medical Center, and Osaka General Medical Center between August 2014 and October 2015. Conventional ultrasonography (US) and MRI were performed to determine ALN status of each patient. When ALN metastasis was suspected, FNAC was performed and the patients with cytology‐proven ALN metastasis were excluded from the study. Patients who had been previously treated with radiotherapy or chemotherapy or who were allergic to eggs were also excluded. A total of 104 patients were recruited, but 4 of them were excluded because SLNs of 2 patients were examined not in frozen sections but with the one‐step nucleic acid amplification (OSNA) method (Sysmex, Kobe, Japan), 1 patient did not undergo US examination, and 1 patient withdrew consent before surgery. Patient characteristics of the 100 patients eventually analyzed for this study are summarized in Table 1. Informed consent was obtained from each patient. This study was approved by the institutional review board of each of the hospitals involved.

**Table 1:** Patient characteristics

	No. of patients
Age, years	
≤50	63
50>	37
Tumor size	
T1	70
T2	30
Tumor histology	
IDC	86
ILC	4
DCIS	6
Other	4
Histological grade	
1	30
2	49
3	17
Unknown	4
Estrogen receptor	
Positive	87
Negative	13
Unknown	0
Progesterone receptor	
Positive	79
Negative	21
Unknown	0
Her‐2/neu	
Positive	18
Negative	75
Unknown	7
Type of surgery	
Lumpectomy	45
Mastectomy	36
SSM, NSM	15
RFA	4

IDC, invasive ductal carcinoma; ILC, invasive lobular carcinoma; DCIS, ductal carcinoma in situ; SSM, skin sparing mastectomy; NSM, nipple sparing mastectomy; RFA, radiofrequency ablation.

### Identification of SLN by Sonazoid and blue dye and/or radiocolloid

After general anesthesia was induced, SNZ (16 *μ*L) in 2 mL saline was injected periareolarly with a 27‐gauge needle, followed by manual massaging of the injection site for about 1–2 min. Contrast‐enhanced lymphatic vessels from the injection site to the axilla were traced by means of US, and the lymph nodes, into which SNZ was draining, were defined as Sonazoid‐enhanced SLNs (SNZ‐SLN). The skin above the SNZ‐SLNs was marked, and the number, shape, size, and location (the depth from the skin and the distance from the lateral edge of the pectoralis major muscle) of SNZ‐SLNs were recorded. Each SNZ‐SLN was then subjected to FNAC with a 22‐gauge needle, followed by SLNB with blue dye and/or radiocolloid (B/R). Periareolar injection of patent blue and indocyanine green was used for 41 patients, periareolar injection of indocyanine green for 40, and periareolar injection of radiocolloid (99mTc‐phytate) and indigocarmine for 19. Periareolar injections of Sonazoid, each dye, and radiocolloid were made at four periareolar sites (3, 6, 9, and 12 o'clock positions around the areola), and each injection was composed of a first intradermal injection of 0.25 mL followed by a second subdermal injection of the same volume (total volume of 2 mL). A total of 37–74 MBq of 99mTc‐phytate was injected 1–4 h before surgery or in the morning of the day before surgery. Each dye was injected immediately before SLNB during surgery. SLN (blue) was defined as a lymph node partially or completely stained by blue dye or directly connected to a blue‐stained afferent lymphatic tract. SLN (radiocolloid) was defined as a lymph node with ex vivo radioactivity (counts per second) measuring 400% or more of that of the axillary background. SLNs identified by blue dye and/or radiocolloid are hereafter abbreviated as B/R‐SLNs. The presence of petechiae left on the surface of most SLNs as the result of examination of each SNZ‐SLN by FNAC was helpful for their identification among the B/R‐SLNs removed by SLNB. If SNZ‐SLNs were not included in the B/R‐SLNs, they were identified by using the information of their location obtained by US before SLNB and by petechiae on their surface. When the intraoperative frozen section analysis of SLNs revealed metastasis, completion ALND was performed; otherwise, only SLNB was performed.

### Ultrasound equipment

US was performed using Logiq E9 with XDclear (GE Healthcare, Tokyo, Japan) and/or Aplio500 (Toshiba Medical Systems, Tokyo, Japan). Both systems were equipped with broadband linear phased‐array transducers that were adapted for harmonic imaging. Contrast‐enhanced scanning was performed with the amplitude modulation method (Logiq E9) or pulse subtraction method (Aplio500) using mechanical index (MI) of 0.2–0.25 for a single focus zone at a depth of 10–50 mm from the surface.

### Histologic examination

At Osaka University Hospital, a 2‐mm thick slice was cut from the middle of each SLN and subjected to intraoperative frozen section examination. The remainder of the SLN as well as the slice subjected to frozen section examination were fixed in 10% buffered formalin, sectioned into 2‐mm slices, and embedded in paraffin. At the other two hospitals, 2‐mm thick slices were serially cut from each SLN and subjected to intraoperative frozen section examination. All the slices were then fixed in 10% buffered formalin and embedded in paraffin. The paraffin sections (4 *μ*m) of these SLNs were subjected to HE staining and immunohistochemistry with an anticytokeratin antibody (AE1/3) (Nichirei, Tokyo, Japan) as described previously 3, 4, 5. For the present study, micro‐ and macrometastases, but not isolated tumor cells (ITCs), were classified as metastases.

### Statistics

Differences among categorical variables were analyzed with a chi‐square test, and differences in the mean values of continuous variables were analyzed with the Student's *t*‐test. *P* < 0.05 was considered significant.

## Results

### Identification of SLN by Sonazoid or blue dye and/or radiocolloid (B/R)

**Figure 1:**
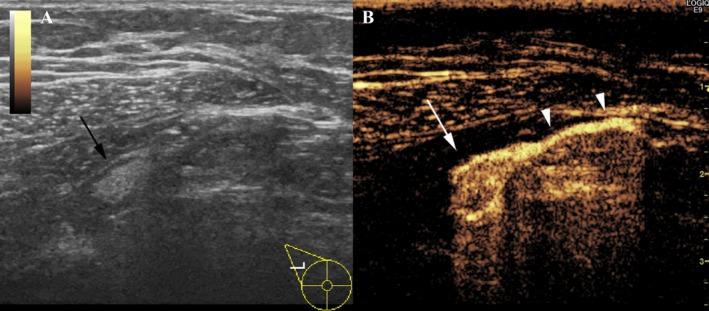
Representative results of dual monitoring of right axillary lymph node basin by Gray‐scale and contrast‐enhanced ultrasound of a patient who received periareolar injection of Sonazoid. (A) Gray‐scale ultrasound image shows elliptical lymph node with fatty hilum (black arrow). (B) Contrast‐enhanced ultrasound image clearly shows that contrast‐enhanced sentinel lymph node (white arrow) is connected to contrast‐enhanced lymphatic vessel (white arrow heads).

A total of 100 patients received the periareolar injection of SNZ and SLNB using B/R. Table 2 shows the number of SLNs per patient identified by SNZ (SNZ‐SLNs) and that identified by B/R (B/R‐SLNs). The identification rate of SLNs was 98% (98/100) for SNZ and 100% (100/100) for B/R. For the next analysis, the two patients with SLNs identified by B/R but not SNZ were excluded. The median number of SLNs identified by SNZ (SNZ‐SLNs) per patient was 1 (mean, 1.52; range, 1–4) and that of B/R‐SLNs (SLNs identified by B/R) was 2 (mean, 2.19; range, 1–5). A significantly lower number of SLNs was detected by SNZ than by B/R (*P* < 0.0001). Representative results of the identification of SNZ‐SLNs by SNZ are shown in Figure 1, demonstrating that SNZ‐SLN, into which SNZ was draining, can be clearly observed. At least one SNZ‐SLN matched B/R‐SLN in all the 98 patients, who had a total of 221 SLNs, 143 of which were identified by both SNZ and B/R, 72 by B/R alone, and 6 by SNZ alone.

**Table 2:** Number of SLNs per patient according to method for the identification of SLNs (blue dye/radiocolloid or sonazoid)

No. of SLNs per patient	No. of patients according to method for the identification of SLNs		*P*
	B/R	SNZ	
0	0	2	<0.0001^a^
1	16	57	
2	52	33	
3	27	6	
4	4	2	
5	1	0	

SLN, sentinel lymph node; SNZ, Sonazoid; B/R, blue dye and/or radiocolloid.

a Student's *t*‐test.

### Metastasis in CE‐SLNs and B/R‐SLNs

**Figure 2:**
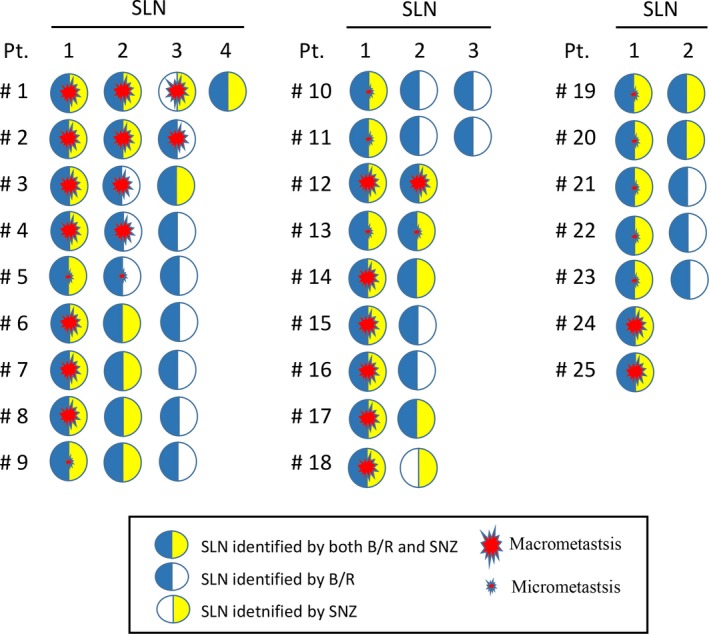
Metastasis in SLNs according to SLN identification method. Twenty‐five patients (Pt. #1–#25) were found to have metastasis in the sentinel lymph nodes (SLN) identified by blue dye/radiocolloid (B/R) and/or Sonazoid (SNZ). These patients had a total of 60 SLNs. Relationship between SLN metastasis (macro‐ or micrometastasis) and the SLN identification method is shown on an SLN‐to‐SLN basis. Blue semicircles indicate that the SLN was identified by B/R, yellow semicircles that the SLN was identified by SNZ, and a half‐blue and half‐yellow circles that the SLN was identified by both methods.

Twenty‐five of the 98 patients had positive SLNs. Detailed information on a node‐by‐node basis for the 60 SLNs obtained from these 25 patients is provided in Figure 2 where metastases in SNZ‐SLNs or B/R‐SLNs for each of the patients are shown on a per‐node basis. The frequency of metastasis was higher for SNZ‐SLNs (73.2%, 30/41) than B/R‐SLNs (56.9%, 33/58), although the difference was statistically not significant (Table 3). Of the 60 SLNs, 39 were identified by both SNZ and B/R, 19 by B/R alone, and 2 by SNZ alone. Metastasis was found in 29 (74.3%) of the 39 SLNs identified by both SNZ and B/R, 4 (21.1%) of the 19 SLNs identified by B/R alone, and in 1 (50%) of the 2 SLNs identified by SNZ alone. Each of the 25 patients with positive SLNs had at least one positive SLN identified by both SNZ and B/R (Fig. 2). On a patient‐by‐patient basis, 25 patients had positive SLNs and 73 had negative SLNs by SNZ, and exactly the same 25 and 73 patients had positive and negative SLNs, respectively, by B/R, indicating a 100% concordance rate between SNZ and B/R for the diagnosis of patients with positive SLNs.

**Table 3:** Metastasis in SLNs according to method for the identification of SLNs in 25 patients with positive SLNs

SLNs identified by	No. of SLNs	No. of positive SLNs	Positivity (%)
B/R	58	33	56.9
SNZ	41	30	73.2
B/R and SNZ	39	29	74.3
B/R, not SNZ	19	4	21.1
SNZ, not B/R	2	1	50.0

SLN, sentinel lymph node; SNZ, Sonazoid; B/R, blue dye and/or radiocolloid.

### FNAC examination of metastases in SNZ‐SLNs

A total of 149 SNZ‐SLNs of all the 98 patients whose SLNs were identified by SNZ were examined by FNAC. Histological examination of these 149 SNZ‐SLNs revealed 20 macrometastases and 10 micrometastases. On an SLN‐by‐SLN basis, sensitivity, specificity, and accuracy of FNAC for all metastases were 33.3%, 99.2%, and 85.9%, respectively, while the sensitivities for macrometastases and micrometastases were 45.0% and 10.0%, respectively (Table 4). On a patient‐by‐patient basis, sensitivity, specificity, and accuracy of FNAC were 28.0%, 98.6%, and 80.6%, respectively, for all metastases, while the sensitivities for macrometastases and micrometastases were 37.5% and 11.1%, respectively.

**Table 4:** Sensitivity, specificity, and accuracy of FNAC for the detection of metastasis in SLNs identified by Sonazoid

	SLN‐by‐SLN basis	Macrometastasis	Micrometastasis
Sensitivity	33.3% (10/30)	45.0% (9/20)	10.0% (1/10)
Specificity	99.2% (118/119)	99.2% (118/119)	99.2% (118/119)
Accuracy	85.9% (128/149)	91.4% (127/139)	92.2% (119/129)
	Patient‐by‐patient basis	Macrometastasis	Micrometastasis
Sensitivity	28.0% (7/25)	37.5% (6/16)	11.1% (1/9)
Specificity	98.6% (72/73))	98.6% (72/73))	98.6% (72/73))
Accuracy	80.6% (79/98)	87.6% (78/89)	89.0% (73/82)

FNAC, fine needle aspiration cytology; SLN, sentinel lymph node.

### Safety

No adverse events related to the periareolar injection of SNZ such as skin reactions around the injection site or allergic reactions were observed immediately or at 3 and 6 months after surgery.

## Discussion

In this study, we have been able to demonstrate that the identification rate of SNZ‐SLN is as high as 98%, which is consistent with the previously reported findings of 75% and 100% by Omoto et al. 13, 18, 93% by Sever et al. 16, and 97% by Cox et al. 17, indicating that identification of SLN by periareolar injection of SNZ is technically simple. In fact, in almost all patients, lymphatic vessels enhanced by SNZ could be easily observed soon after periareolar injection, after which the SLN into which SNZ was draining could also be clearly identified. Although ultrasound contrast agents have the same architecture, consisting of microbubbles and a capsule, SNZ is believed to be most chemically stable in the body due to its extremely high uniformity in diameter 19. Such outstanding stability is thought to make a longer lasting US examination possible than can be realized with the other second‐generation contrast agents, thus leading to an easier identification of SNZ‐SLNs.

The mean number of SNZ‐SLN (1.52) was significantly lower than that of B/R‐SLN (2.19) (*P* < 0.0001). Omoto et al. also reported that the mean number of SNZ‐SLNs was 1.1, which was also smaller than that of radiocolloid‐SLNs (1.8) 13. The median diameter of SNZ is ranged from 2.4 to 3.5 *μ*m, which is larger than that of any dye or radiocolloid. The behavior of tracers for use in SLNB is strongly depends on their particle size. A large particle tracer does not pass into the lymphatic system easily, but once trapped in the lymph node, it is retained for a relatively long time and the risk of travelling into many non‐SLNs is low. The larger particle size of SNZ may partly explain the significantly lower mean number of SLNs obtained with SNZ than with the B/R method.

While it remains controversial how many SLNs should be removed to accurately predict ALN status 20, 21, the mean number of removed SLNs has reportedly been between 1.2 and 3.4 (range: 1–8) for patients who underwent conventional SLNB using B/R 22, 23. SLN is defined as the first lymph node in a regional nodal basin that receives lymphatic flow from the primary tumor. Theoretically, therefore, there should be only one SLN in most patients. However, it is generally accepted that more than one SLN should be removed to reduce the false‐negative rate 24, because it has been suggested that not all B/R‐SLNs are necessarily true SLNs but that some of them might be secondary LNs receiving lymphatic flow from the true SLNs. For the 25 patients in our study with positive SLNs, SNZ‐SLNs showed a tendency toward a higher frequency of metastasis than B/R‐SLNs (73.2% vs. 56.9%). Taken with the fact that the number of SNZ‐SLNs is smaller than that of B/R‐SLNs, it can be speculated that SNZ‐SLNs represent the true SLNs more accurately than B/R‐SLNs. This speculation seems to be supported by the observation that SNZ can actually visualize the SLNs into which SNZ is draining through the lymphatic vessels. SNZ‐SLNs thus identified are more likely to be true SLNs than those detected by B/R. With SLNB using blue dye, blue‐stained lymphatic vessels which connect to the SLNs are often observed. In most patients, more than one blue‐stained SLN is removed, but usually no attention is paid to which of these SLNs connect to the blue‐stained lymphatic vessels on histological examination. Thus, if positivity for metastasis of SLNs connecting and those not connecting to the blue‐stained lymphatic vessels is compared, it might turn out to be more frequent in the former than the latter.

It is also very important to accurately identify the SNZ‐SLNs intraoperatively. In the present study, an SNZ‐SLN was localized by US, and its location was recorded in details, such as the depth from the skin, as well as its shape, size, and the distance from the lateral edge of the pectoralis major muscle. Tracing the blue dye‐stained lymphatic tract was also helpful for detection of SNZ‐SLNs. In addition, most of SNZ‐SLNs showed petechiae on their surface as a result of repeated FNAC. Using these findings thus made it relatively easy to identify the SNZ‐SLNs. However, placement of a clip within the SLN 25, 26 or injection of a dye into the SLN 27, 28 has been reported as a more accurate method for the intraoperative identification of LNs which have been detected preoperatively. In a future study, SNZ‐SLNs should therefore be marked preoperatively with a clip or dye under US guidance.

Since each of the 25 patients with positive SLNs had at least one positive SNZ‐SLN (Fig. 2), SNZ‐SLN is considered to be a reasonable target for FNAC to detect metastasis. We also assessed how accurately FNAC could detect metastases in SNZ‐SLNs and found that its sensitivity, specificity, and accuracy were 33.3%, 99.2%, and 85.9%, respectively. Findings for sensitivity of CNB for contrast‐enhanced SLNs have been reported as 53% by Cox et al. 17., and as 65% by Sever et al. 16, indicating that CNB is superior to FNAC in the detection of metastases in SNZ‐SLNs. Cox et al. also suggest that CNB can predict the absence of extensive axillary disease, information which should be very useful for deciding whether to omit ALND 17. However, the accuracy of CNB is still not high enough for CNB to replace SLNB. Such an insufficient sensitivity might stem from the heterogeneity of tumor cells in an SLN. However, sensitivity could be improved by more aggressive and repeated sampling by CNB. This possibility needs to be further investigated.

In this study, detection of SLNs by contrast‐enhanced ultrasonography was done under general anesthesia and immediately followed by SLNB/ALND and surgery of the breast since detection of SNZ‐SLNs was experimental so that the patient's distress should be minimized. However, if the sensitivity of CNB were improved in future, CNB of SNZ‐SLNs would better be done under local anesthesia in advance to the surgery because the preoperative determination of SLN status is very useful in the planning of the following surgical and systemic treatment. Furthermore, it may lead to the avoidance of SLNB for patients with negative SNZ‐SLNs.

In this study, no skin reactions around the periareolar injection site nor allergic reaction were observed, demonstrating that SNZ is safe when injected periareolarly even though it has been developed as an intravenous agent. SNZ consists of microbubbles and a capsule made of phosphatidylserine of egg yolk origin, so that SNZ is contraindicated for patients with an episode of egg allergy. Two other studies on the periareolar injection of SNZ for identification of SLNs have also reported that no serious side effects were observed 13, 18.

We used a 27‐gauge needle for the injection of Sonazoid due to the following reasons. First, although a 22‐gauge needle was used for the injection of Sonazoid in the initial studies by Omoto et al. 13, 18, Sever et al. used a thinner needle (26 gauge) for the injection for SonoVue. In spite of the difference in the gauge of needles, similarly high identification rates of SLN were reported. Second, in our previous studies, dye and/or radiocolloid were injected into the dermis and subdermis of the periareolar using a 26‐ or 27‐gauge needle for facilitating them to drain into the lymphatics 3, 4, 5. We think such a fine injection into the dermis and subdermis can be easily done with a 27‐gauge needle. Besides, the diameter of microbubbles in Sonazoid (median: 2.6 *μ*m) and SonoVue (median: 2.5 *μ*m) is so small that the usage of a 27‐gauge needle is unlikely to affect the stability of the microbubbles and to compromise their ability to visualize SLN. In fact, we were able to show a high identification rate of SLN of 98% in the present study.

In conclusion, the present feasibility study has demonstrated that identification of SLNs by the periareolar injection of SNZ followed by US is technically simple and can attain an SLN identification rate as high as 98%, which is comparable to that obtainable with conventional B/R. Moreover, it appears that SNZ‐SLNs represent the true SLNs which are the first to receive lymphatic flow from the tumor, and thus seem to be a reasonable target for the use of FNAC for the detection of metastasis in SLNs. However, the accuracy of FNAC was lower than that reported for CNB which seems to be a more suitable method for the detection of metastases in SNZ‐SLNs. Our preliminary results for the detection of SNZ‐SLNs need to be validated by a future study comprising a larger number of patients.

## Conflict of Interest

Dr. Noguchi has received research funding and honoraria from Daiichi‐Sankyo, Co., Ltd. for work performed outside of the current study. Drs. Shimazu and Ito have received honoraria from Daiichi‐Sankyo, Co., Ltd. for work performed outside of the current study.
